# Rv2231c, a unique histidinol phosphate aminotransferase from *Mycobacterium tuberculosis*, supports virulence by inhibiting host-directed defense

**DOI:** 10.1007/s00018-024-05200-8

**Published:** 2024-05-02

**Authors:** Sheeba Zarin, Mohd. Shariq, Nilisha Rastogi, Yashika Ahuja, P. Manjunath, Anwar Alam, Seyed Ehtesham Hasnain, Nasreen Zafar Ehtesham

**Affiliations:** 1 Institute of Molecular Medicine, Jamia Hamdard, Hamdard Nagar, New Delhi, India; 2 Department of Life Science, School of Basic Sciences and Research, Sharda University, Greater Noida, Uttar Pradesh 201310 India; 3 Cell Signaling and Inflammation Biology Lab, ICMR-National Institute of Pathology, New Delhi, 110029 India; 4 Department of Biotechnology, School of Engineering and Technology, Sharda University, Greater Noida, 201310 India; 5 Department of Biochemical Engineering and Biotechnology, Indian Institute of Technology, New Delhi, 110016 India

**Keywords:** Apoptosis, Histidine biosynthesis, M2 macrophage polarization, Necroptosis, Pyroptosis, TLR4

## Abstract

Nitrogen metabolism of *M. tuberculosis* is critical for its survival in infected host cells. *M. tuberculosis* has evolved sophisticated strategies to switch between de novo synthesis and uptake of various amino acids from host cells for metabolic demands. Pyridoxal phosphate-dependent histidinol phosphate aminotransferase-HspAT enzyme is critically required for histidine biosynthesis. HspAT is involved in metabolic synthesis of histidine, phenylalanine, tyrosine, tryptophan, and novobiocin. We showed that *M. tuberculosis* Rv2231c is a conserved enzyme with HspAT activity. Rv2231c is a monomeric globular protein that contains α-helices and β-sheets. It is a secretory and cell wall-localized protein that regulates critical pathogenic attributes. Rv2231c enhances the survival and virulence of recombinant *M. smegmatis* in infected RAW264.7 macrophage cells. Rv2231c is recognized by the TLR4 innate immune receptor and modulates the host immune response by suppressing the secretion of the antibacterial pro-inflammatory cytokines TNF, IL-12, and IL-6. It also inhibits the expression of co-stimulatory molecules CD80 and CD86 along with antigen presenting molecule MHC-I on macrophage and suppresses reactive nitrogen species formation, thereby promoting M2 macrophage polarization. Recombinant *M. smegmatis* expressing Rv2231c inhibited apoptosis in macrophages, promoting efficient bacterial survival and proliferation, thereby increasing virulence. Our results indicate that Rv2231c is a moonlighting protein that regulates multiple functions of *M. tuberculosis* pathophysiology to increase its virulence. These mechanistic insights can be used to better understand the pathogenesis of *M. tuberculosis* and to design strategies for tuberculosis mitigation.

**Supplementary Information:**

The online version contains supplementary material available at 10.1007/s00018-024-05200-8.

## Introduction

*Mycobacterium tuberculosis* (*M.tb*), is an intracellular pathogen which cause Tuberculosis (TB) and is highly contagious and gets transmitted through infected individuals. *M.tb* primarily infects human lungs and causes classic pulmonary TB disease. It also causes extrapulmonary TB, which affects other organs and tissues such as the lymph nodes, brain, kidneys, and spine [1]. *M.tb* is the deadliest infectious disease killer, surpassing SARS-CoV 2 [2]. The COVID pandemic while creating lot of challenges and severely impacting global efforts to end TB, has posed lessons to be learnt to eradicate TB [3–5]. TB treatment require a long regimen of multiple drugs that has toxic side-effects, which is the major reason for non-compliance of chemotherapy among patients that leads to evolution of *M.tb* into drug-resistance strains [6]. Chemotherapies that are shorter and easier to administer are urgently required to treat TB. An estimated 10.6 million people were affected with TB in 2021, an increase in more than 4% as compared to 2020. From 2015 to 2021, the net reduction was 5.9%, one-sixth of the first milestone of the WHO End TB strategy. As per the WHO, nearly 450,000 new cases of rifampicin resistant TB were reported in 2021 which underlines the increasing trend of drug resistant TB worldwide [2]. The COVID19 pandemic, which has dramatically fuelled the disruption of essential TB services, has hampered efforts to eliminate TB as a global public health threat [7]. It is astounding that no effective vaccine is available for a microbe identified almost a century and a half ago, which has caused widespread suffering and death [8]. An effective vaccination strategy and faster, easier, and more effective chemotherapy interventions are urgently needed to achieve the 2035 milestones for TB elimination. Although the pathogen and disease have evolved significantly, there are still many blind spots that prevent us from fully understanding them. Sequencing data accumulates faster than the amount of information that can be annotated. It has been postulated that up to half of the expected coding sequences are misannotated or encode functions different from those originally predicted [9]. In contrast, nearly 33% of the reported enzymatic activities does not relate with the coding sequences [10]. Several mechanistic features of *M.tb* pathophysiology remains unexplored and hence there is an exigent need to examine the role of uncharacterized genes and proteins.

Transposon mutagenesis has predicted that the *M. tb Rv2231c* gene is essential for growth in the spleens of *M. tb*-infected C57BL/6J mice [11], however its functional role has not been characterized yet. Sequence analysis predicted that Rv2231c contains a pyridoxal phosphate (PLP)-binding domain belonging to the family of (PLP)-binding aminotransferases [12]. This is similar to the histidinol-phosphate aminotransferase (HspAT) of *Mycobacterium bovis * (*M. bovis*). Proteomic analyses showed that Rv2231c is one among the abundant proteins in *M. tb* [13]. A previous study has demonstrated its overexpression under drug and starvation conditions. Rv2231c showed tenfold higher expression in macrophages after 24 h of infection [14]. Rv2231c is predicted to be a key drug target that can be exploited to control *M.tb*. The dubious annotation of Rv2231c, its ability to establish infection, its relative abundance, the presence of a PLP-binding domain, and its genomic organization adjacent to the signature sequence Rv2231A make it a potential therapeutic target [15].

The present study demonstrated that Rv2231c is an HspAT in *M.tb*. It is required for axenic proliferation and survival of *M.tb* in mouse macrophages. Recombinant *Mycobacterium smegmatis* (*M. smegmatis*) expressing Rv2231c suppressed proinflammatory cytokines, thereby pointing to its role as a virulence factor in the bacilli. Rv2231c is a potent TLR4 agonist involved in the regulation of proinflammatory cytokine secretion. Rv2231c regulates macrophage apoptosis to modulate host–pathogen interactions, which is essential for maintaining chronic infection and causing spread and pathogenesis. These findings suggest that Rv2231c is a metabolic modulator of His synthesis that regulates His-dependent nitrogen metabolism in *M.tb*, which is required for efficient survival, vigorous proliferation, and pathogenicity in infected hosts. Interestingly, our results also suggest that it is an essential metabolic regulator that possesses multiple functions that dampen host defenses to increase virulence.

## Materials and methodology

### Bacterial strains, media, reagents, and culture conditions

*M. smegmatis* MC^2^155 cells obtained from ATCC, USA, were cultured on Middlebrook 7H9 media (BD Biosciences). *E. coli* DH5 and *E. coli* BL21 DE3 cells were grown in Luria–Bertani (LB) media supplemented with 30 µg/ml kanamycin. Cultures of *M. smegmatis* and *E. coli* were grown at 37 °C in a shaker incubator, set at 160 and 200 rpm, respectively. Isopropyl-*β*-d-1-thiogalactopyranoside (IPTG) sarcosine, 3-(4,5-dimethylthiazol-2-yl)-2,5-diphenyltetrazolium bromide (MTT), kanamycin, imidazole and staurosporine were purchased from Merck. Cell culture media and reagents, including DMEM, GlutaMAX and fetal bovine serum (FBS), and antibiotic-antifungal solution were obtained from Gibco, USA. Antibodies were obtained from BD Biosciences and Cell Signalling Technology (USA).

### Cell culture and growth conditions

RAW264.7 murine macrophage cells, TLR2 knockout RAW264.7 cell (RAW-ΔTLR2), TLR4 knockout RAW264.7 cell (RAW-ΔTLR4) and TLR2/TLR4 double-knockout RAW264.7 cell (RAW-ΔTLR2/4), obtained from BEI resources, USA, were cultured in DMEM complete media consisting of 10% FBS, 1% GlutaMAX and 1% antibiotic-antifungal reagent; and incubated at 37 °C maintained at 5% CO_2_ level.

### Phylogenetic study

The Mycobrowser (https://mycobrowser.epfl.ch/genes/Rv2231c) was used to extract the protein sequence of H_37_Rv *Rv2231c*. A phylogenetic tree of Rv2231c was constructed using pairwise BLAST alignment. The BLAST was done to retrieve the Rv2231c protein sequences of Mycobacteria from the KEGG database [16] and used in “Phylogeny.fr” to construct an unrooted evolutionary tree [17]. The similarity between Rv2231c of *M. tb*H_37_Rv and other Mycobacteria was inferred from the pedigree.

### In silico structural and functional analysis

Various computational approaches have been used to elucidate the possible roles of this hypothetical protein. The conserved domain database [18] and PROSITE were used for the domain and motif searches. The percentage of disorder was estimated using ANCHOR [19] and PONDR^®^ VSL2 software [20]. Subcellular protein localization was predicted using DeepLoc 2.0 [21]. Semi-quantitative protein abundance data were generated using PaxDB 4.0 [13].

### Cloning, expression, and purification of Rv2231c from *M. tuberculosis*

The *M.tb* H_37_Rv genomic DNA was obtained from the BEI Resources. *Rv2331c* gene was PCR-amplified using set of primers that contained 6x His-tag and restriction enzyme sites (Table S1). The pET28a (Addgene) vector was used to clone the *Rv2231c* gene using *Bam*HI and *Eco*RI restriction sites. Recombinant construct having the *Rv2231c* gene was transformed into endotoxin-free Clear Coli (BL21-DE3) cells. Rv2231c protein expression was carried out by inducing the recombinant cells with 1 mM IPTG for 4 h at 37 °C. Rv2231c protein purification was done using the Ni–NTA affinity chromatography in Tris-NaCl buffer (100 mM NaCl, 20 mM Tris–HCl, pH 8.0) and dialysis was carried out overnight at 4 °C in 10% glycerol-Tris-NaCl buffer. Endotoxin contamination in purified Rv2231c protein was assessed using the Limulus amebocyte lysate assay kit (Thermo Fisher Scientific). The protein samples incubated at 37 °C for 2 h showed no endotoxin contaminants. The concentration of purified Rv2231c protein was evaluated using Bradford's reagent (Bio-Rad).

### Construction of *M. smegmatis* knock-in

The pST-Ki expression vector was used to develop a knock-in of Rv2231c in *M. smegmatis* [22]. *Bam*HI and *Eco*RI restriction sites were used to subclone Rv2231c from pET28a into pST-Ki. Transformation-competent *M. smegmatis* cells were electroporated with pST-Ki containing Rv2231c in a 0.2 cm cuvette at 2500 V, 1000 Ω resistivity, and 25 F capacitance. Positive clones of recombinant *M. smegmatis* (*Ms*_Rv2231c) were grown on Middlebrook 7H11 agar plates (BD Biosciences) supplemented with kanamycin (20 µg/ml), 10% OADC, and 0.5% glycerol. The positive clones expressing Rv2231c was further confirmed using western blot analysis incorporating anti-rabbit αRv2231c antibody [23].

### Production of polyclonal anti-rabbit antibody against Rv2231c

Purified Rv2231c protein (His tag at N-terminus) was used to induce anti-rabbit polyclonal antibody. The first dose was administered to a 90-day old New Zealand rabbit with purified recombinant Rv2231c protein (500 µg) emulsified in 500 µl Freund’s incomplete adjuvant (500 µg recombinant Rv2231c protein + 500 µl adjuvant in PBS pH 7.4). Booster doses of purified Rv2231c were administered three times at interval of 28-days. Blood sample was collected from ear vein of rabbit post 10 days since last booster. The serum was separated from the blood and frozen until further use.

### Subcellular localization of Rv2231c

Various *M.tb*-cell fractions, including whole-cell lysate, culture filtrate, cell membrane, and cell wall, were obtained from BEI Resources. Thirty micrograms of each fraction were loaded on SDS-PAGE, and probed for presence of Rv2231c by western blotting using Rv2231c antibody [24].

### Biophysical characterization of recombinant Rv2231c

#### Oligomer state determination by size-exclusion chromatography (SEC) and dynamic light scattering (DLS)

Purified Rv2231c was analyzed for various oligomer states by SEC using a Superdex 75 column equipped with AKTA purifier (GE Healthcare). The column was 1st equilibrated with Tris-buffer containing 100 mM NaCl and 20 mM Tris–HCl (pH 8.0). The protein was eluted, and the concentration of each protein fraction was assessed by measuring the absorbance at 280 nm. The molecular mass of purified Rv2231c protein was assessed by SDS-PAGE and compared with the standard M.W. plot. Xtal concept spectrosize-300 was used to calculate the hydrodynamic radii of Rv2231c [25]. Residual impurities from the protein sample (1.0 mg/ml) were removed using a 0.2 µm PVDF-membrane filter. The hydrodynamic radius of Rv2231c was determined using a preloaded software.

#### Secondary structure determination using circular dichroism (CD) and FTIR spectroscopic techniques

Purified Rv2231c (0.30 mg/ml) was dissolved in Tris-buffer (100 mM NaCl and 20 mM Tris–HCl pH 8.0). Circular dichroism (CD) spectroscopic analysis was performed using a Jasco 1500 CD spectropolarimeter. A quartz cuvette of path length 1 mm was used for the CD measurements. Continuous scanning (20 nm/min) from 240 to 200 nm was performed. Data were collected and processed using the K2D2 software after each sample was scanned three times [26]. A concentrated Rv2231c protein sample (4 µl) was spotted onto a diamond ATR reflective element. An Agilent Cary 600 instrument with a DTGS detector was used to acquire FTIR spectra with a resolution of 4 cm^−1^. The absorption mode was set at 128 scans to collect Rv2231c spectra at room temperature compared to the 128 scan single beam spectra of the buffer alone. The second derivative of the spectrum was calculated using MATLAB (MathWorks Inc., Massachusetts, USA) for kinetic applications. The spectrum shows no evidence of water vapor above 1750 cm^−1^. The band amplitudes of the spectra were normalized and the baseline was fitted using a least-squares curve-fitting program. The secondary structure of Rv2231c was deduced by fitting the curve with amide I region. The second-derivative spectrum was used to locate the peak positions using the Voigt function [27].

#### Thermal stability of Rv2231c determination by differential scanning calorimetry (DSC) and CD

The thermal stability and melting temperature (Tm) of Rv2231c were analyzed using DSC (MicroCal). Protein samples (1 mg/ml) were placed in Tris-buffer. Then Rv2231c protein sample was loaded in a sample cell and heated from 25 to 80 °C. Thermograms (Origin Lab Corporation, Northampton, MA, USA) were analyzed using Origin software. The results obtained were confirmed using CD over the temperature range of 25–90 °C. Molar ellipticity was calculated as previously described [28].

#### Structural modeling of Rv2231c

The 3D model of the Rv2231c protein was revealed using an alpha-fold 2 protein structure database and a web server [29]. Alpha-Fold 2 predicted a highly accurate structure using evolutionarily related sequences and multiple sequence alignment (MSA). The PDB structural model was used to analyze the secondary structures. The stereochemical quality of the Rv2231c-model was verified using PROCHECK software [30]. The PDB file was visualized using the PyMol Protein Molecular Visualizer.

### Spectrophotometric analysis of Rv2231c histidinol-phosphate aminotransferase activity

The biochemical activity of purified Rv2231c protein was assessed by measuring the conversion of NAD ^+ ^to NADH using a spectrophotometer at 340 nm. The conversion of Histidinol phosphate and ketoglutarate into l-glutamate and imidazole acetol phosphate, respectively is not measurable spectrophotometrically. A secondary reaction was therefore carried out using Glutamic acid dehydrogenase (Sigma Aldrich) to convert the product of initial reaction (l-glutamate) into a reactant (2-oxoglutarate) in presence of cofactor NAD^+^. The increase in NADH production during the course of secondary reaction was monitored spectrophotometrically at 340 nm wavelength [31]. Increase in absorbance indicated that the secondary reaction was progressing, confirming the functionality of the recombinant Rv2231c protein. The standard assay reaction solution consisted of Tris–HCl buffer prepared using Tris–HCl (50 mM) pH 8.0, NAD^+^ (250 mM), NaCl (150 mM), histidinol phosphate (2000 mM), pyridoxal-5-phosphate (20 mM), glutamate dehydrogenase (2.37 U), 2-oxoglutarate (1000 mM) and 1 mM Rv2231c [32].

### Uptake and survival assays for Mycobacteria

RAW264.7 macrophages (0.25 × 10^6^ cells/well) were seeded in 24-well plate and cultured overnight in DMEM media at 37 °C. The cells were then infected with *Ms*_Vc (*M. smegmatis* containing vector) and *Ms*_Rv2231c (*M. smegmatis* containing Rv2231c) at a MOI of 1:10. After 4 h of infection, RAW264.7 cells were gently washed thrice with 1 x PBS. The infected RAW264.7 cells were resuspended briefly in DMEM containing gentamycin (50 µg/ml) to kill the extracellular bacteria. The infected RAW264.7 cells were lysed with SDS (0.1%), cell lysate containing intracellular bacilli was diluted appropriately and plated on LB agar plates and incubated at 37 °C for 72 h. The number of intracellular bacilli was enumerated at 24, 48 and 72 h using CFU assay [33]. The live bacteria (*Ms*_Vc or recombinant *Ms*_Rv2231c) obtained in the cell lysate were labelled with FITC (0.1%) and the bacterial uptake in the RAW264.7 cells were assessed flow cytometrically.

### Estimation of cytokines, reactive oxygen species (ROS), and nitric oxide (NO) production, and analysis of macrophage surface markers and apoptosis

RAW264.7 macrophage cells (0.25 × 10^6^ cells/well) were infected with *Ms*_Vc and *Ms*_Rv2231c, as described above. The uninfected control RAW264.7 cells as well as infected cells were washed, incubated for 24 h and 48 h in complete DMEM containing gentamicin (50 µg/ml). The levels of secreted proinflammatory cytokines (TNF, IL-6 and IL-12) in culture supernatant were estimated using ELISA kit, as per the manufacturer’s protocol (BioLegend). Nitric oxide (NO) concentrations were estimated by adding 50 µl of Griess reagent to 150 µL of the supernatant and incubating in dark for 30 min. The NO concentrations were determined by recording the absorbance at 545 nm [34]. ROS, activation markers, and apoptosis were analyzed in RAW264.7 cells collected in 1 x cold PBS with 2% FBS. 20 µM CM-H_2_DCFDA (Thermo Fisher Scientific) was used to quantify the ROS [35]. FITC-Annexin V kit (BD Biosciences) for Apoptosis detection was used to measure apoptosis. For flow cytometric assessment of macrophage activation markers, cells were treated with an F_c_ block for 15 min at 4 °C. Cells were then treated with cocktail of fluorescently labelled anti-mouse antibodies specific for cell-surface markers and incubated at 4 °C for 15 min [36]. After staining procedure, cells were gently washed with FACS buffer followed by fixing in 4% paraformaldehyde. Antibody-labelled cells (50,000) were then acquired using LSR Fortessa FACS (BD biosciences) and data was analyzed using FlowJo software.

### Immunofluorescence analysis of the Rv2231c-TLR (Toll-like receptor) interaction

0.2 × 10^6^ macrophage cells (TLR2-knockout, TLR4-knockout, and RAW264.7) were seeded on sterile coverslips placed in a 24-well plate and incubated for 4 h. Cells were treated with purified Rv2231c protein (4 µg/ml) for 2 h and fixed with formaldehyde solution (4%). The fixed cells were then subjected to washing thrice with PBS and incubated in presence of antibody (1:3000) raised against Rv2231c. After 2 h, antibody coated cells were treated with Alexa Fluor 594-conjugated anti-rabbit secondary antibody (1:1000) and incubated for 1 h. The antibody-labelled cells on the coverslip were treated with Prolong antifade glass mountant and cells were visualized under a confocal microscope [37].

### Western blot analysis

Various purified fractions of *M. tuberculosis* (30 μg of whole-cell lysate, culture filtrate, cell membrane and cell wall) were treated with 2.5 x SDS sample loading buffer consisting of 25% glycerol, SDS (5%), Tris–HCl (0.125 mM) pH 6.8), bromophenol blue (0.1%), and 100 mM dithiothreitol. Samples were heated at 95 °C for 10 min. Proteins were run on SDS-PAGE and then transferred to a polyvinylidene difluoride (PVDF) membrane using a semi-dry blotting system (Bio-Rad Laboratories). The proteins on the PVDF membranes were assessed by treatment with primary antibodies against Rv2231c, GroEL2, and Mpt32, followed by the addition of HRP-conjugated secondary antibodies (Sigma). The presence of proteins on the PVDF membranes was detected using Enhanced Chemiluminescence Substrate (ECL) (DSS Takara Bio India Pvt. Ltd.) to develop signals [38] and images were acquired using a ChemiDoc system (Bio-Rad).

### Statistical analysis

Data obtained from experiments were analyzed using one-way and two-way ANOVA. The mean standard deviation (SD) was calculated for the readings acquired from three set of experiments. GraphPad Prism 9 was used to analyze the data obtained. The values where *p* ≤ 0.05, were considered statistically significant.

## Results

### Phylogenetic analysis revealed that Rv2231c is only found in pathogenic Mycobacteria

**Fig. 1:**
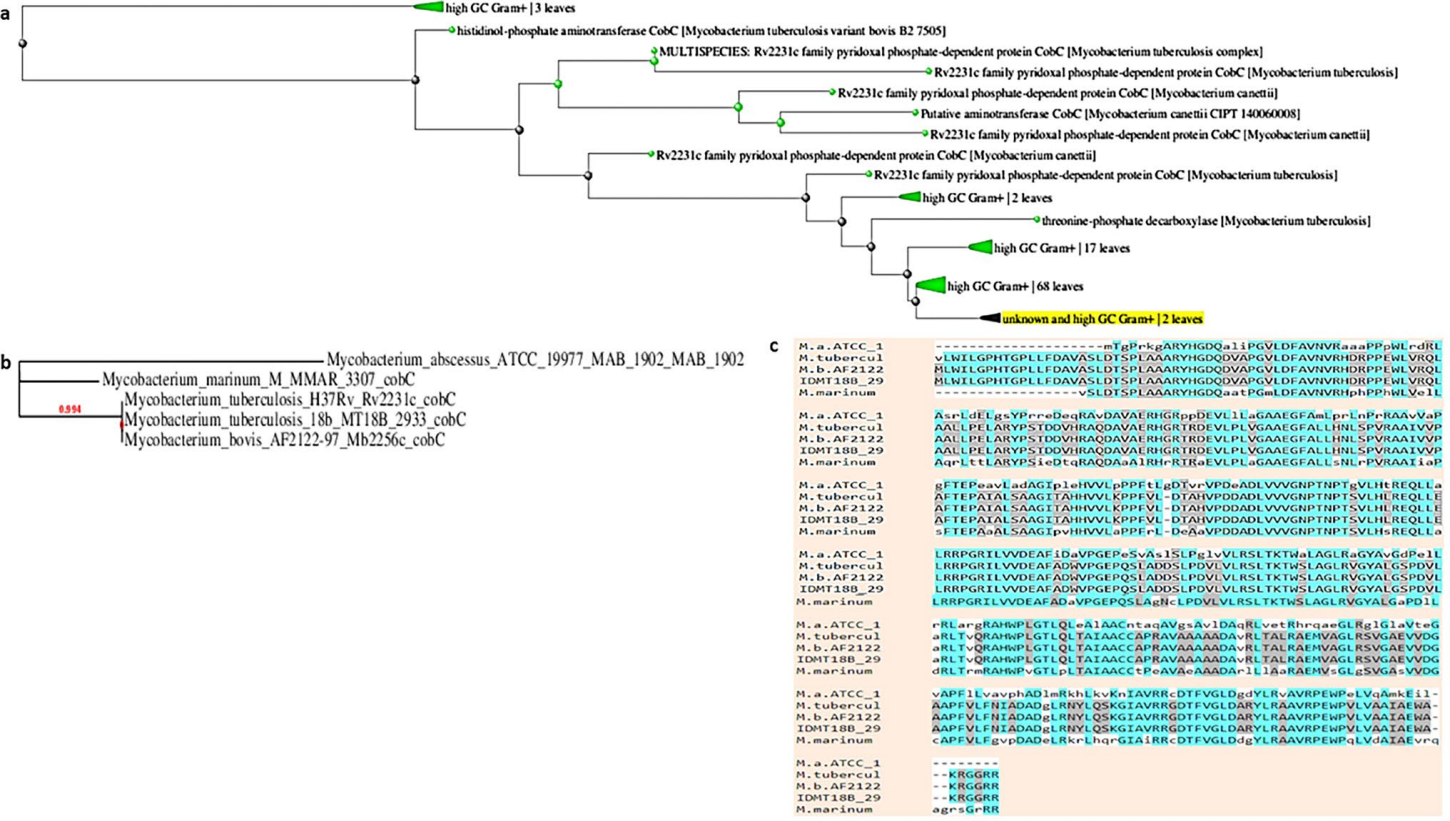
Rv2231c is a unique pathogenic Mycobacterial protein. **a** A BLAST tree view, constructed using BLAST pairwise alignment and the fast minimum evolution tree approach, showed homology between *M. tb* and *M. bovis* HspAT. **b** A phylogenetic tree of *M. tb*-Rv2231c showing closely related proteins from different mycobacterial species. The number of substitutions per site was directly correlated with branch length in the tree. (**c**) Sequence alignment of *M. tb-*Rv2231c with related Mycobacterial species revealed 100% identity with *M. bovis*. The most conserved residues are highlighted in similar colors using BLOSUM62.M.a.ATCC_1: *M. abscessus*, M.tubercul: *M. tuberculosis*, M.b.AF2122: *M. bovis,* IDMT18b_29: *M. tuberculosis* 18b

To gain insight into the evolutionary relationship between Mycobacterial HspAT, the top 100 identity hits, including *M. tb* H_37_Rv Rv2231c (CobC), were analyzed using BLAST. BLAST tree showed similarities between HspAT and Mycobacterial species (Fig. 1a). One representative of each Mycobacterial species was selected, and the amino acid sequences were submitted to Phylogeny.fr to generate a rootless tree. The length of the branches of the rootless tree represented the evolutionary distance among homologous proteins in various mycobacterial species (Fig. 1b). In addition, sequence alignment of pathogenic Mycobacterial Rv2231c was performed using BLOSUM62 to gain evolutionary insight. The result showed that *M. tb* H_37_Rv Rv2231c had 100% similarity with *M. bovis*. It was distantly related to *Mycobacterium marinum* (*M. marinum*) and *Mycobacterium abscesses* (*M. abscesses*) (Fig. 1c). BLAST results also showed that 91% of the amino acid residues were conserved (amino acid residues with similar colors were the most conserved) (Fig. 1c). Therefore, our results suggest that *M.tb* Rv2231c is an evolutionarily conserved protein retained in pathogenic Mycobacteria, implying its functional importance in the regulation of pathogenesis.

### Rv2231c is a stable pyridoxal-phosphate-dependent aminotransferase in *M. tb*

**Fig. 2:**
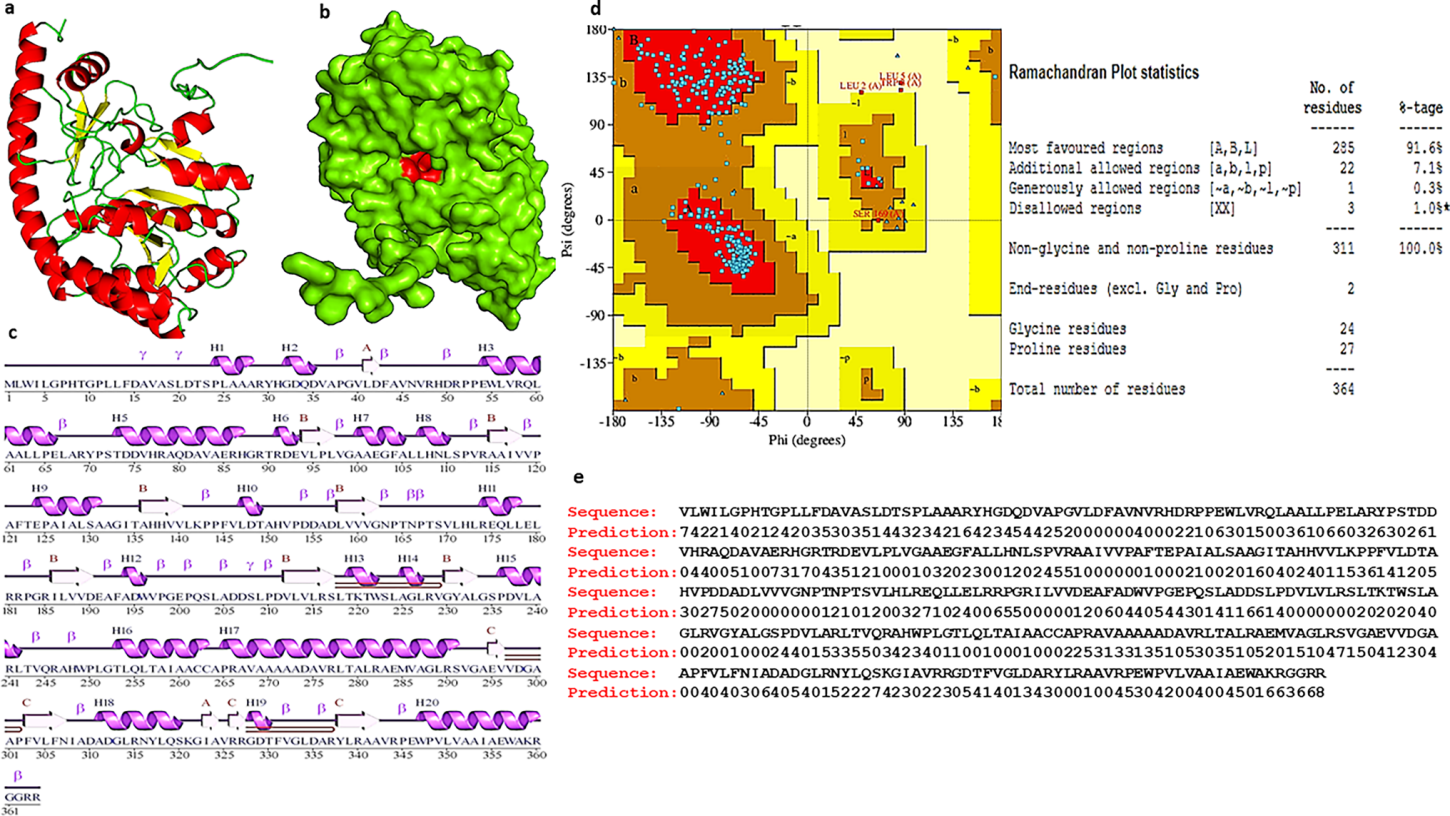
Structural analysis of the Rv2231c protein using AlphaFold. **a** and **b** Cartoons predicting the tertiary structure of *M. tuberculosis* Rv2231c from the alpha-fold. **c** The secondary structure of Rv2231c contains 20 α-helices, 13 β-strands, three β-sheets, three β-hairpins, two β-α-β units, 18 helix-helix interactions, 26 β-turns, and four γ-turns. **d** Ramachandran plot of Rv2231c showing the allowed and disallowed amino acid regions. **e** Predicted solvent accessibility of amino acid residues of Rv2231c. Scores ranged from 0 (buried residues) to 8 (heavily exposed residues)

Since no high-resolution structure has been reported for the uncharacterized Rv2231c, we generated a structural model using AlphaFold to decipher the domains and motifs present in *M. tb*-Rv2231c (Fig. 2a). We used Interpro-Scan for domain analysis, which indicated the presence of a conserved aminotransferase domain spanning 42–332 amino acids and containing catalytic and pyridoxal phosphate-binding sites (Fig. 2b). The generated model was highly accurate, with a confidence level of over 90%. Further structural analysis revealed that Rv2231c contains 3-beta sheets of 13 beta strands and 20 alpha helices (Fig. 2c). The structure was relatively stable, except for the very flexible N-terminus. The Ramachandran plot showed that 91.6% of the amino acid residues belonged to the preferred regions, whereas 7.4% and 1% were found in the allowed and disallowed regions, respectively (Fig. 2d). The results confirmed that the constructed structural model was of high-quality. In addition, we constructed a solvent accessibility map, which showed that 36% of the residues were deeply buried, 26% were lightly exposed, 29% were moderately exposed, and 7% were heavily exposed (Fig. 2e). In conclusion, our structural analyses revealed that Rv2231c has a stable configuration containing conserved catalytic and pyridoxal phosphate-binding sites, suggesting that it is a functional enzyme with HspAT activity.

### Rv2231c is a functional aminotransferase in *M. tb*

**Fig. 3:**
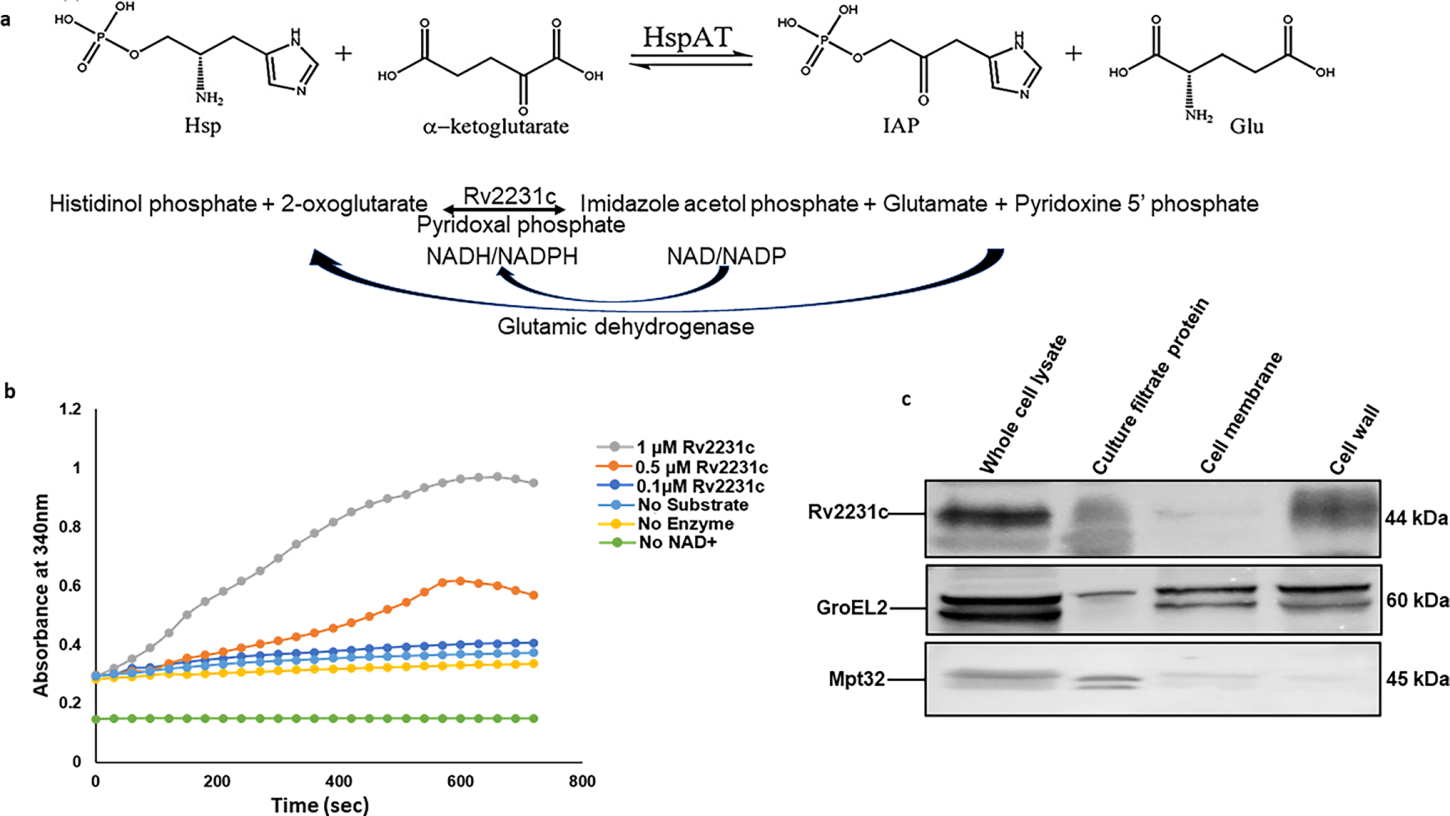
*M. tb* Rv2231c is a functional HspAT. **a** Schematic representation of the GDH-coupled assay system showing the two steps used to measure the Rv2231c aminotransferase activity. **b** Activity of purified recombinant *M. tb-*Rv2231c was assessed at 340 nm and an increase in absorbance was recorded over time. This increase in absorbance was attributed to the increased conversion of NAD^+^ to NADH. An enzyme-free reaction was used as a negative control (**c**) Western blot analysis of different purified fractions of *M. tb* showed that Rv2231c is localized to multiple sites in *M. tb*. GroEL2 and Mpt32 localization was considered as control

A significant class of amino acid biosynthetic enzymes include aminotransferases that utilize the vitamin B6-derived coenzyme pyridoxal-5-phosphate (PLP) typically following ping-pong bi-bi reaction mechanism. HspAT, an essential enzyme for biosynthesis of histidine, catalyzes the transfer of an amino group from L-histidinol phosphate (Hsp) to 2-oxoglutarate that lead to formation of glutamate and imidazole acetol phosphate (IAP). Initially, PLP gets converted to pyridoxamine-5-phosphate (PMP3) and in later phase of reaction, recognition and catalysis of amino acid substrates occurs. HspAT activity of Rv2231c can be studied by using a reaction mixture that contains excess of reactants and adding limiting concentration of enzyme. Table S2 lists the names of the reactants used. Spectrophotometric measurement of the increased absorbance at 340 nm confirmed the production of NADH. This showed that Rv2231c of *M. tb* exhibited HspAT activity (Fig. 3a and b). Localization study using various purified fractions of *M. tuberculosis* and Rv2231c specific antibody revealed its presence in culture filtrate (CFP) and cell wall fractions (Fig. 3c)*.* Additionally, the steady-state kinetics of transamination activity of Rv2231c was monitored using a two-stage aminotransferase assay [39]. Our results revealed that Rv2231c showed excellent transaminase activity toward its known substrate, Hsp. Rv2231c exhibits a specific activity of 8.9333 mol · min^−1^ · mg^−1^. The catalytic rate of Rv2231c was measured at various Hsp concentrations. Our results indicated that Rv2231c exhibited standard Michaelis–Menten kinetics (Table S3). The catalytic efficiency (k_cat_/K_M_) of Rv2231c for Hsp was (0.72 ± 0.02)10 ^6^ M^−1^S^−1^. We observed the Michaelis constant (K_M_) and k_cat_, which were 0.6 ± 0.05 mM and (4.33 ± 0.07)10^2^ S^−1^, respectively. Our results suggest that *M. tb* Rv2231c is a functional HspAT exhibiting Michaelis–Menten kinetics.

### Rv2231c exists as a folded globular monomer in the solution

**Fig. 4:**
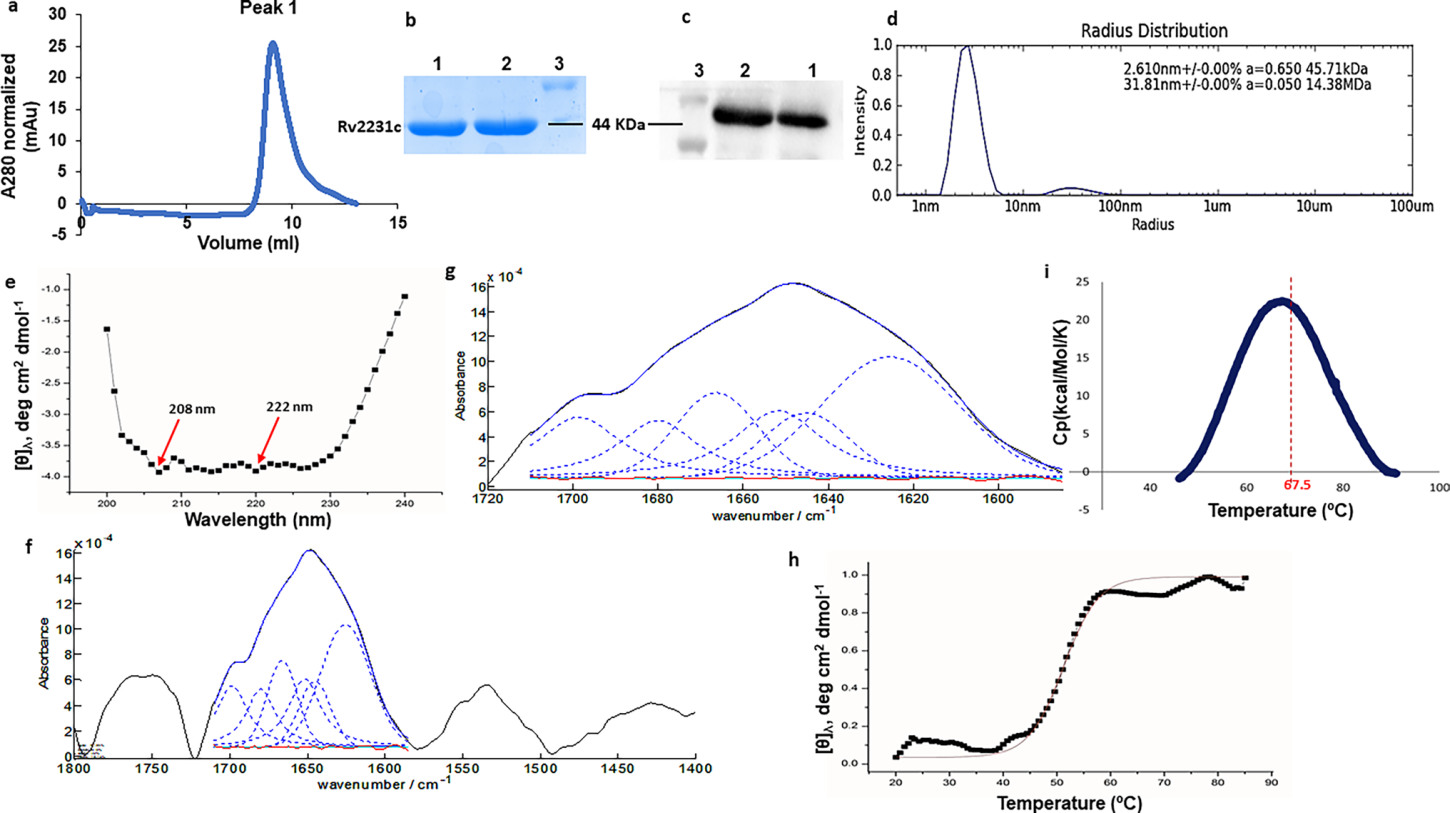
*M. tb* Rv2231c is a thermostable protein present as a folded globular monomer in solution. **a** Analytical gel filtration chromatography (GFC) chromatogram of Rv2231c; blue curve represents the elution profile of Rv2231c. **b** and **c** SDS-PAGE and western blot analysis of Rv2231c after Ni–NTA and gel filtration chromatography. Lane 1: Rv2231c obtained after GFC; lane 2: affinity-purified Rv2231c; Lane 3: protein size markers (BLUeye prestained protein ladder). **d** Size distribution analysis of purified Rv2231c. Hydrodynamic radii were determined using Dynamic Light Scattering. **e** Far-UV CD spectrum of Rv2231c. Purified Rv2231c was loaded into a quartz cuvette, and spectra were measured at 200–250 nm. The secondary structure content was analyzed using K2D2 software. **f** and **g** FTIR spectrum of Rv2231c. Each protein sample (1 mg/ml) was scanned using a Pro MID/FAR IR instrument (Agilent Technologies) in the spectral range of 800 to 3800 cm^−1^. **h** and **i** The thermal transition curve of Rv2231c was estimated by CD and DSC spectroscopy

Biophysical characterization of Rv2231c protein is required to gain better insight into its biochemical activity and to correlate its structure and function. We examined the homogeneity of Rv2231c in solution to estimate its molecular weight and folding states. DLS (Dynamic Light scattering) and SEC/GFC (Size Exclusion Chromatography/Gel Filtration Chromatography) were used to determine the size and shape of Rv2231c [40, 41]. Our results showed that Rv2231c eluted as a single peak at an elution volume of approximately 10 ml during analytical GFC, indicating that Rv2231c was present as a monomer in the solution (Fig. 4a). The peak fraction was concentrated using a 10 kDa Centricon filter and subjected to SDS-PAGE and western blotting (Fig. 4b and c). These results suggested that Rv2231c migrated as a single band at 44 kDa. This was consistent with the elution pattern obtained in a fully calibrated Superdex 75 (10 × 300) with ovalbumin of molecular weight 44 kDa eluted at a 10 ml elution volume [42]. To gain further insight into the molecular structure of Rv2231c, the hydrodynamic radius was calculated using DLS. The size distribution of the protein samples revealed a single species with a molecular weight of approximately 45 kDa (Fig. 4d). The R.H. value of 2.610 nm agreed with the DLS value (2.8 nm) for ovalbumin (44 kDa). Taken together, our (SEC)/GFC and DLS findings suggest that Rv2231c has a folded spherical shape with a molecular weight of approximately 44 kDa.

### Secondary structure and thermostability of Rv2231c

CD and FTIR spectroscopy were used to determine the structural features of Rv2231c. The presence of helical structures in the protein was evident from presence of negative peaks at 208 and 222 nm in the far-UV CD spectra [43] (Fig. 4e). CD data analysis showed that Rv2231c contains 28.84% helices and 13.66% sheets. This finding was further verified by FTIR spectroscopy. The FTIR data showed absorbance in the 1700–600 cm^−1^ range caused due to amide I vibration upon stretching of C = O bond from the protein backbone (Fig. 4f and g). The secondary structural details of Rv2231c were calculated using the amide I vibration with a 25% helix and 19% sheet [44]. The Rv2231c protein-matched amide I bands are shown in (Fig. 4f and g). The visible bands were denoted as alpha helices near 1660 cm^−1^, random coils near 1678 cm^−1^ and 1690 cm^−1^, beta sheets near 1650 cm^−1^, and loops near 1620 cm^−1^. The secondary structure contents obtained by CD and FTIR spectroscopy are shown in (Table S4). Our results suggest that Rv2231c has a well-organized and stable structure.

In addition, we analyzed the thermostability of Rv2231c by CD. A drop in ellipticity at 220 nm was used to monitor the thermal denaturation of Rv2231c with rise in temperature from 25 to 80 °C [45]. The curves started to deviate from 45 °C, and Tm was calculated to be 57 °C (Fig. 4h). This observation was further supported by differential scanning calorimetry (DSC), which analyzed the heat-initiated phase transitions of Rv2231c. DSC was used to quantify H-bonding and other thermodynamic parameters. The DSC calculated Tm was equivalent to 67.5 °C (Fig. 4i). The Tm values of Rv2231c calculated using CD, DSC, and thermal denaturation profiles were very similar (Table S5). Our results suggest that Rv2231c is a thermodynamically stable protein, with a Tm of approximately 65 °C.

### Rv2231c is a unique aminotransferase secreted by *M. tb*

Our in silico analysis suggested that Rv2231c belongs to aminotransferase class I family of PLP-dependent proteins (Suppl. Fig. 1a). Cellular localization of Rv2231c was predicted using DeepLoc 2.0, which revealed the cytoplasmic and secretory nature of Rv2231c (Suppl. Fig. 1b). To confirm its cellular localization in *M. tb*, cell-fraction proteins were separated by SDS-PAGE and probed with Rv2231c antibody using western blot method. Our results showed that Rv2231c was mainly present in the CFP (culture filtrate) and cell wall fractions. A small amount of Rv2231c was detected in the cell membrane (Fig. 3c). In addition, we also analyzed the abundance of Rv2231c in the *M. tb* proteome using PaxDB 4.0 protein abundance database. Rv2231c is moderately expressed in *M. tb* and belongs to the bottom 25% of the most abundant proteins (Suppl. Fig. 1c). Furthermore, our protein–protein BLAST analysis showed that *M. tb* Rv2231c CobC is identical to *M. bovis* Mb2256c CobC. However, when we used the αRv2231c antibody to detect the expression of Mb2256c CobC in *M. bovis* whole-cell lysate, we unexpectedly observed no signal corresponding to CobC, implying that CobC is not expressed in *M. bovis*. These findings suggest that Rv2231c is localized to multiple locations in *M. tb* and may play multiple roles in regulating *M.tb* physiology and pathogenesis.

### Rv2231c facilitates Mycobacterial survival and virulence in macrophages

Based on our previous results, we hypothesized that Rv2231c maybe a crucial protein in *M. tb* that might play an essential role in its virulence and pathogenicity. To confirm our hypothesis, in vitro examination of presence of recombinant *Ms*_Rv2231c in the macrophages was done and compared with *Ms*_Vc.

**Fig. 5:**
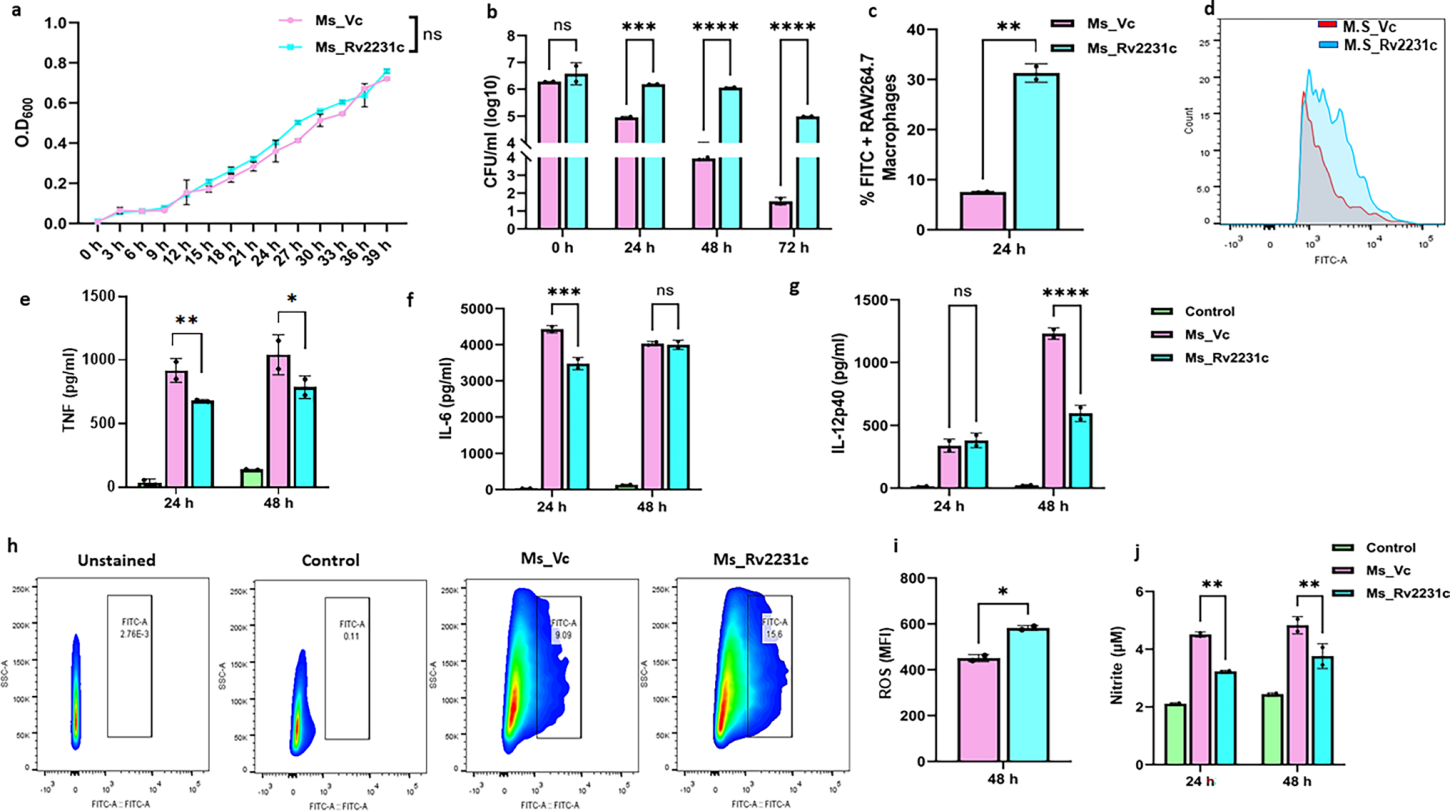
*M. smegmatis* expressing *M. tb*-Rv2231c protein showed higher intracellular survival in RAW264.7 macrophage cells and inhibits secretion of inflammatory cytokines. Macrophage cells were infected with FITC-labelled *Ms*_Vc and *Ms*_Rv2231c at MOI of 1:10 and incubated for 4 h. The cells were washed to remove extracellular bacteria and replenished with DMEM. **a** Growth kinetics of *M. smegmatis* expressing Rv2231c or vector-only cells. The 7H9 Middlebrook broth was inoculated with primary cultures (OD 0.01) of *Ms*_Vc or *Ms*_Rv2231c and incubated at 37 °C at 200 rpm. OD_600_ nm was measured at intervals of 3 h to 40 h. **b** Enumeration of phagocytosed and surviving bacteria at 0, 24, 48, and 72 h post infection was carried out using CFU assay. **c** and **d** Flow cytometry analysis of bacterial survival in macrophages 24 h after infection. **e–g** Estimation of pro-inflammatory cytokines upon infection. RAW264.7 cells (0.25 × 10^6^ cells/well) were infected with *Ms*_Vc and *Ms*_Rv2231c at MOI of 1:10. ELISA was done to measure the cytokines TNF-α, IL-6, and IL-12p40 in cell culture supernatants collected 24 and 48 h after infection. **h** FACS plot showing the ROS production after 48 h. **i** Bar graph showing ROS production levels. **j** The NO content was estimated spectrophotometrically at 545 nm using Griess reagent. Standard deviation (SD) was calculated using the means of two independent experiments. Graphs were created with the help of GraphPad software. *p ≤ 0.05, **p ≤ 0.01, ***p ≤ 0.001, ****p ≤ 0.0001, ns; not significant

*Ms*_Vc and Ms_Rv2231c showed similar growth patterns *invitro* (Fig. 5a) CFU was determined at 0 h post-infection, which showed no difference in the bacterial count of *Ms*_Vc or *Ms*_Rv2231c phagocytosed in RAW264.7 cells. This indicated that Rv2231c does not play a role in increasing invasiveness of recombinant *Ms*_Rv2231c. Interestingly, however, we observed significantly increased survival of *Ms*_Rv2231c in macrophages compared to *Ms*_Vc after 24 and 48 h of infection (Fig. 5b). In addition, we confirmed our results using FACS analysis (Fig. 5c and d). These observations suggest that Rv2231c enhances the survival of *Ms*_Rv2231c in macrophages.

### Rv2231c inhibits macrophage activation and production of proinflammatory cytokines to dampen innate defense

Rv2231c protein is found on the surface of the cell and is secreted in *M.* tb, thereby regulating host–pathogen interactions. The host pro-inflammatory immune response modulates the outcome of infection. The levels of proinflammatory cytokines secreted by RAW264.7 macrophage cells after infection with *Ms*_Vc, or *Ms*_Rv2231c was assessed to gain insight into immune modulation by *M. tb* Rv2231c. *Ms*_Vc and *Ms*_Rv2231c induced secretion of IL6 and TNF. At 24 h, there was a decrease in TNF and IL-6 level secreted by macrophages infected with *Ms*_Rv2231c. However, the decrease In TNF at 48 h was lower than that at 24 h. We did not observe any changes in the levels of IL-6 secreted after 48 h of infection (Fig. 5e and f). A significant inhibition of IL-12p40 secretion in macrophages infected with *Ms*_Rv2231c was seen at 48 h of infection (Fig. 5g). These results highlight that Rv2231c inhibits the generation of a pro-inflammatory response that can be exploited by *M. tb* to evade the host's innate immune defenses.

Key antimicrobial effectors, including ROS and RNS, were measured to assess the ability of Rv2231c to dampen host protective responses. ROS levels were increased in *Ms*_Rv2231c-infected macrophages compared to *Ms*_Vc (Fig. 5h and i). Interestingly, macrophages infected with *Ms*_Rv2231c produced reduced NO levels in contrast to *Ms*_Vc-infected macrophages (Fig. 5j).

**Fig. 6:**
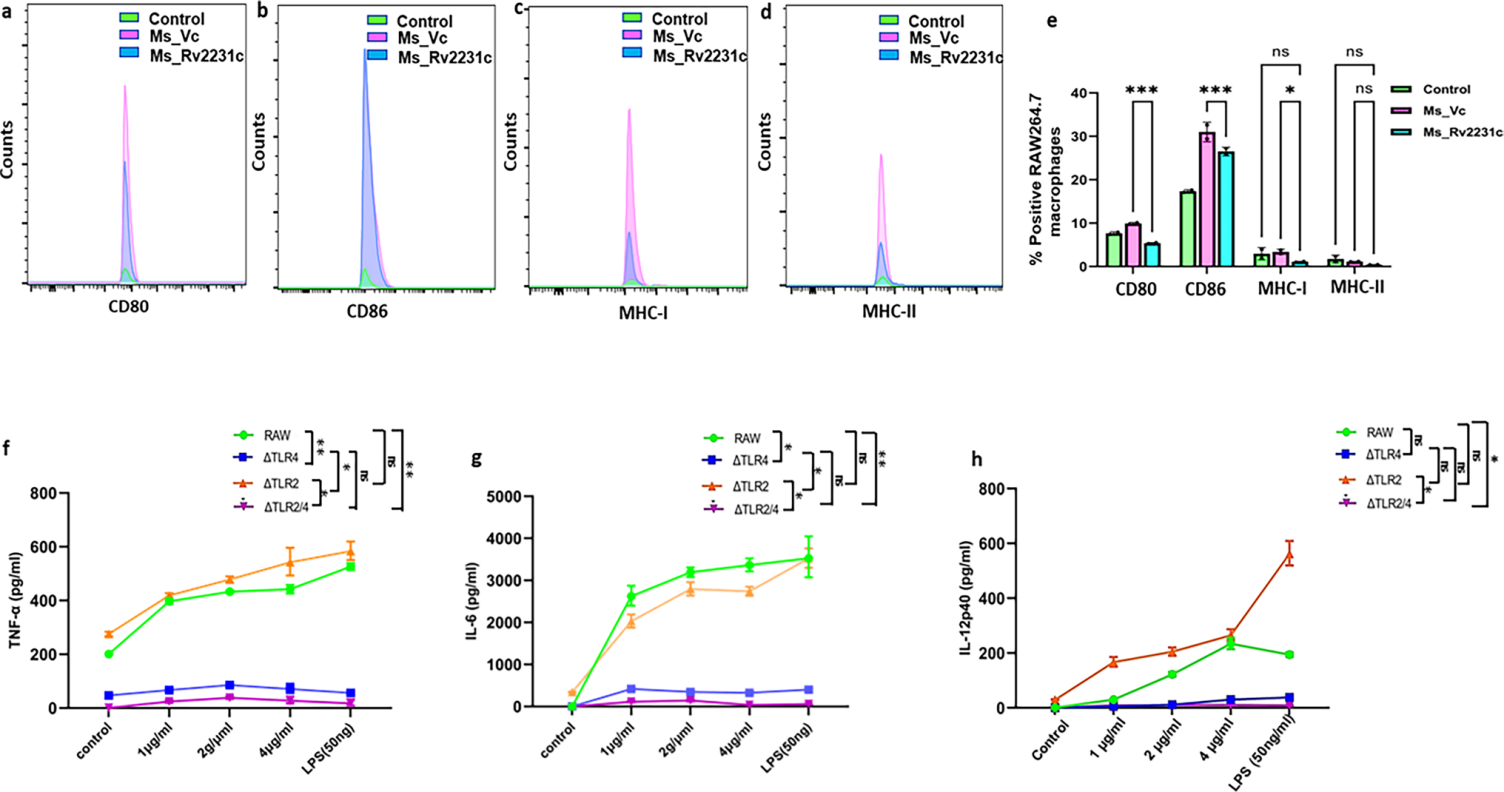
Rv2231c inhibits M2 macrophage activation. RAW264.7 macrophage cells were infected with *Ms*_Vc and *Ms*_Rv2231c at MOI of 1:10. Infected cells were harvested after 48 h, stained with fluorescent tagged antibodies CD80, CD86, MHC-I, and MHC-II, and analyzed using flow cytometry. The culture supernatant from 48 h was collected to estimate the NO concentrations. Recombinant *M. smegmatis* expressing Rv2231c suppressed antigen presentation. FACS data show percent of RAW264.7 cells infected with *Ms*_Vc and *Ms*_2231c expressing **a** CD80, **b** CD86, **c** MHC-I, and **d** MHC-II. **e** Flow cytometry histogram showing the expression levels of CD80, CD86, MHC I, and MHC II. **f**–**h** RAW264.7, RAW-ΔTLR2, and RAW-ΔTLR4 knockout cells were stimulated with Rv2231c (1, 2, and 4 µg/ml) and LPS (50 ng/ml) for 48 h. TNF, IL-6, and IL-12p40 levels were estimated by ELISA. Standard deviation was calculated using the means of two independent experiments. All graphs were created using GraphPad software. *p ≤ 0.05, ***p ≤ 0.001, ns; not significant

Next, we determined the potential of Rv2231c to regulate macrophage function. We analyzed the level of expression of CD80, CD86, MHC-I, and MHC-II, markers for activated macrophage. The histogram was generated incorporating a consistent cell count of 50,000 for all samples, a quantity chosen to ensure statistical robustness given the reported low expression percentages of CD80, CD86, and MHC-II on RAW264.7 cells in prior investigations [46, 47]. In Fig. 6a–d, specifically panels a, b, c, and d, the evaluation of CD80, CD86, MHC-I, and MHC-II expression levels was conducted through the mean fluorescence intensity (MFI). The curves overlaid in control, *Ms*_Vc, or *Ms*_Rv2231c-infected cells illustrate variations in the Y-axis, reflecting two contributing factors: (i) alterations in the overall RAW264.7 cell population, indicated as % positive in Panel e of Fig. 6, and (ii) dynamic fluctuations in per-cell expression of CD80, CD86, MHC-I, and MHC-II, as denoted by MFI values. Consequently, while control cells exhibited lower curve heights, those treated with *Ms*_Vc or *Ms*_Rv2231c displayed elevated curves. This observation aligns with findings from previous studies [46, 47], wherein RAW264.7 cells under LPS treatment, manifested pronounced alterations in the expression of these markers. The height variations in the curves thus reflect both the shifts in overall cell population and the dynamic changes in individual marker expression, substantiating the significance of the observed outcomes. Hence, it is concluded that macrophages infected with *Ms*_Rv2231c exhibited lower expression of CD80, CD86, and MHC-I as compared to *Ms*_Vc-infected cells. There was no significant differences in expression of MHC-II. These results suggest that *M.tb* uses Rv2231c to inhibit macrophage activation, which is required for efficient immune responses.

### Rv2231c is recognized by TLR4 to regulate downstream signaling cascades

Many *M. tb* proteins are recognized by TLR2 (ESAT-6) or TLR4 (3- and 4-acylated lipomannan, HSP60, and HSP65) [48]. To investigate the role of Toll-like receptors (TLRs) in regulating the secretion of pro-inflammatory cytokines in response to the Rv2231c protein, we conducted experiments using RAW264.7 macrophages and TLR knockout cell lines, including RAW-ΔTLR2, RAW-ΔTLR4, and RAW-ΔTLR2/4. These cells were treated with varying concentrations (1, 2, or 4 µg) of purified Rv2231c protein. The results demonstrated that ΔTLR2 and RAW264.7 cells exhibited a notable increase in the secretion of IL-6, IL-12, and TNF-α in response to Rv2231c protein treatment. In contrast, ΔTLR4 and ΔTLR2/4 cells did not show significant secretion of these cytokines (Fig. 6f–h). These findings suggest that the absence of TLR4 on the macrophage surface impairs their ability to recognize Rv2231c, resulting in lack of induction of pro-inflammatory cytokine secretion. These findings imply that TLR4 plays a crucial role in detecting Rv2231c at the macrophage cell surface to initiate downstream signaling cascades that produce inflammatory cytokines.

To further validate the involvement of the innate host receptor in recognizing Rv2231c and the downstream signaling pathways, we treated RAW264.7 cells, ΔTLR2, and ΔTLR4 knockout macrophage cells with purified Rv2231c protein. Immunofluorescence microscopy was used to study the localization of Rv2231c on macrophage cell surfaces. Following treatment of RAW264.7 cells, ΔTLR4, and ΔTLR2 with Rv2231c protein, anti-Rv2231c antibody was used to study the localization of Rv2231c on the macrophage cell surface. Rv2231c protein was seen to localize on the cell surface, binding to its cognate receptor in RAW264.7 and ΔTLR2 knockout cells, whereas Rv2231c specific signal was absent in ΔTLR4 cells, indicating that Rv2231c is recognized by TLR4 at the macrophage cell surface (Suppl. Fig. 2e).

To strengthen these findings, we conducted protein docking experiments using a modelled Rv2231c protein. The docking results showed an interaction between Rv2231c and the ligand-binding site of TLR4 (Suppl. Fig. 2a–d). These findings collectively suggest that Rv2231c functions as a specific TLR4 agonist, playing a pivotal role in the regulation of pro-inflammatory cytokine secretion.

### Recombinant *M. smegmatis* expressing Rv2231c inhibits macrophage apoptosis

**Fig. 7:**
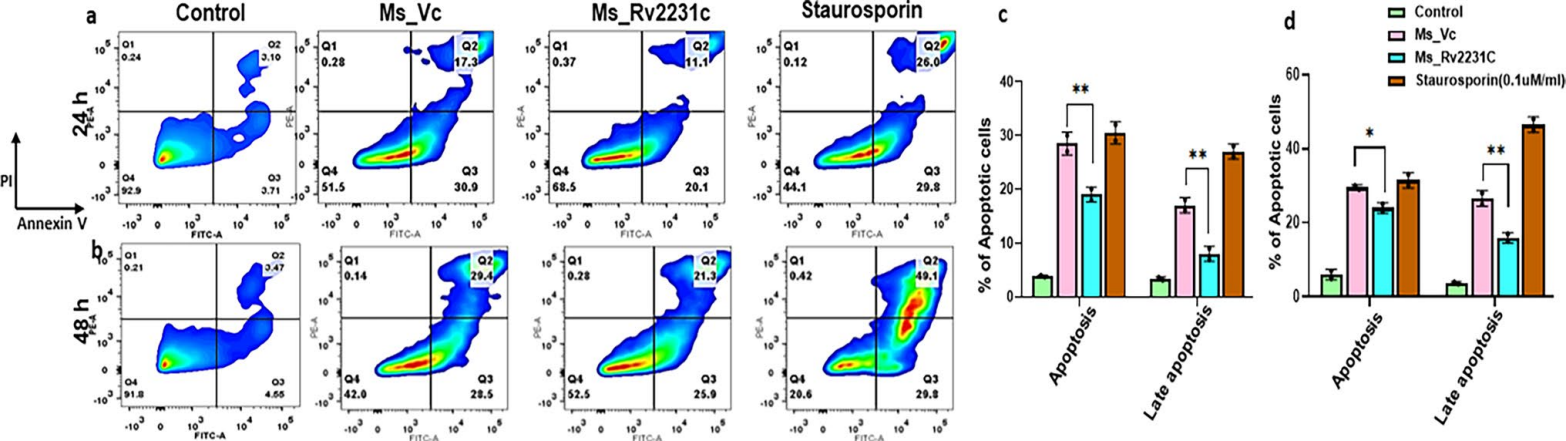
Recombinant *M. smegmatis* containing Rv2231c inhibits apoptosis of RAW264.7 cells. RAW264.7 cells were infected with *Ms*_Vc and *Ms*_Rv2231c at MOI of 1:10. At 24 and 48 h post-infection, the cells were stained with the Annexin V/PI kit, and apoptosis was analyzed. FACS analysis of different stages, that is, early and late apoptosis in Uninfected Control group, *Ms*_Vc-and *Ms*_Rv2231c infected murine macrophages **a** 24 h and **b** 48 h of infection along with staurosporine stimulation as a positive control. The bar graph shows the early and late apoptotic cells in different samples after 24 h and 48 h, as shown in Figure (**c** and **d**)

MTT assay was used to study the effect of Rv2231c protein on the viability of RAW264.7 cells. RAW264.7 cells were cultured in presence of purified Rv2231c protein for 24 and 48 h. Our results showed that treatment of RAW264.7 with Rv2231c (8 µg/mL) induced cell death after 48 h (Suppl. Fig. 3a). This led us to examine the role of Rv2231c in modulating one of the critical cell death pathways, such as apoptosis. *Ms*_Rv2231c and *Ms*_Vc infected along with uninfected control RAW264.7 macrophage cells were labelled with Annexin V/Propidium Iodide (PI) and the percent of apoptotic cells were analyzed using flow cytometry. Our results showed that Rv2231c protein of *M.tb* could inhibit apoptosis in macrophage cells (Fig. 7a–d).

### Discussion

Rv2231c (CobC) encodes a protein which has been implicated in synthesis of cobalamin. However, de novo synthesis of cobalamin in *M. tb* remains controversial [49]. It appears that Rv2231c plays multiple roles in *M. tb* pathophysiology to dampen host defense. CobC has been shown to act as a threonine phosphate decarboxylase in *Pseudomonas aeruginosa (P. aeruginosa)*, and plays an important role in generation of 1-amino-2-propanol phosphate. CobC aid in transport of heme and studies on *Salmonella enterica* shows that it also acts as alpha-ribazole phosphatase.

BLAST analysis of *M. tb*-Rv2231c protein showed the closest homology to *M. bovis* HspAT. *M. bovis* harbors an ortholog of Rv2231c annotated as Mb2256c, which could be a possible aminotransferase (CobC). However, CobC expression was not detected in *M. bovis* using anti-Rv2231c antibody. A very weak homologue of Rv2231c is present in *M. smegmatis*, which encodes a divergent protein that was also not detected using antibody generated against Rv2231c of *M. tb*. Global protein similarity was analyzed using Clustal Omega and the results showed that *M. smegmatis* CobC possesses only 24% identity, whereas protein blast shows similarity between 150 and 240 amino acids. Therefore, in this study, we utilized non-pathogenic, fast-growing *M. smegmatis* as a surrogate model for *M. tb*, to study the virulence-causing mechanism of Rv2231c protein. *M. smegmatis* is more amenable to genetic manipulation techniques, such as gene expression studies and can be easily grown in the BSL1 facility compared to *M. bovis*, which is a slow-growing mycobacterium [50].

A close look at the STRING protein–protein interaction data of CobC homologs from different organisms revealed that most of the interacting partners of CobC from *P. aeruginosa* and *Salmonella enterica* belonged to the cobalamin pathway (Suppl. Fig. 3d–f). Surprisingly, in *M. tb*-Rv2231c, all interaction partners originated from the histidine biosynthetic pathway. This prompted us to investigate its function as HspAT. *M. tb* possesses enzymes that are essential for production of all the 20 amino acids [51]. Enzyme-synthesizing essential amino acids are potential therapeutic drug targets for TB [52]. These enzymes are either pathway-specific or have different catalytic capacities, with functional overlap between cellular processes. A recent study suggested using TyrAT from *Leishmania infantum* to develop an anti-*Leishmania* drug [53]. The results of Tropical Disease Research’s drug target prediction imply that the enzymes of the histidine pathway of *M. tb* contain no homologs in human [54]. A genome-wide mutagenesis study revealed that several genes encoding enzymes in the histidine biosynthetic pathway are essential for the survival of *M. tb* [55].

The host deprives *M. tb* of essential amino acids to control infection, as generic mechanism observed for other pathogens as well. For example, the host cells deprive Chlamydia and Leishmania of tryptophan, in order to eliminate infection caused by these tryptophan-auxotrophs [56, 57]. During *M. tb* infection, the host cells upregulate production of histidine ammonia-lyase (HAL) and histidine decarboxylase (HDC) for degrading freely available histidine. As a counter-response, *M. tb* induces increased de novo synthesis of histidine in order to evade the host response [58]. Therefore, if the intracellular *M. tb* can import free histidine in sufficient amount from the host cell that can suffice its physiological requirements, histidine biosynthetic enzymes would be redundant. Interestingly, *M. tb* completely lacks histidine utilization system (Hut) that comprises of genes encoding histidine-degrading enzymes and histidine transporters [59]. In contrast, environmental non-pathogenic *M. smegmatis*, possesses a functioning Hut system [60]. These variations in histidine-dependent metabolism between environmental non-pathogenic mycobacteria and obligate-pathogenic mycobacteria could provide vital evidences in understanding the effect of selection pressures in modulating *M. tb* metabolism during pathogenicity and host tropism. Notably, the *M. tb* genome encodes two HspATs, HisC1 and HisC2, whose expression decreased 1.7-fold upon starvation. Rv2231c exhibiting the same HspAT activity was previously shown to undergo tenfold increase in expression during macrophage infection [14]. These findings indicate the need for continuous de novo histidine synthesis to persist within the infected host to maintain virulence and pathogenesis.

It is important to highlight that Rv2231c is part of the *M. tb* secretome. We showed that *M.tb*-Rv2231c is a TLR4 agonist which is recognized by the ligand-binding pocket of TLR4 present on macrophage cells. This was evident as the surface localization of Rv2231c was not observed in mouse macrophage cells which lacked TLR4 receptor (RAW-ΔTLR4 cells). The binding of *M. tb*-Rv2231c with TLR4 evokes downstream signaling that modulate the secretion of cytokines TNF, IL-6 and IL-12, which are critical components of the immune system in defense against *M. tb*. Increased production of TNF, IL-6 and IL-12 lead to activation of macrophage and increased expression of activation markers on surface of cells. Our results showed an overall reduction in secretion of TNF, IL-6 and IL-12p40 after infection of RAW264.7 macrophage cells with *Ms*_Rv2231c. However, at 48 h post infection there was no decrease in IL-6 levels but a significant decline in IL-12p40. The observed opposing trends in IL-6 and IL-12p40 secretion upon infecting RAW cells with recombinant *M. smegmatis* reflects upon the discernible functions of IL-6 and IL-12 across various stages of the immune response to *M.tb* infection. IL-6, being a pleiotropic cytokine, predominantly participates in early inflammatory processes and attains peak expression during the initial phases of infection [61]. In contrast, IL-12, which is indispensable for the development of adaptive immunity, may be secreted at later stages of infection and may not exhibit concurrent expression with IL-6. Rv2231c inhibits the expression of CD80, CD86, and MHC-I. Therefore, we hypothesize that Rv2231c promotes M2 macrophage polarization, which prevents the robust generation of defense against *M. tb*. *M. tb* also suppresses cell death mechanisms for intracellular survival, replication and in modulating cytokine secretion. It was fascinating to observe the regulatory effect of Rv2231c on cell death mechanism such as apoptosis. Macrophage cells infected with *M. tb* commonly activates cell death mechanisms- necrosis which causes cell lysis, and apoptosis in which cell membrane remains intact [62]. Apoptosis-induced cell death in macrophages that are infected with *M. tb* results in killing of mycobacteria without eliciting inflammation and efficiently induces T cell responses via enhanced antigen presentation. On the other hand, necrosis promotes the unbridled spread of the pathogen and surpasses the activation of the adaptive immune response. Avirulent *M. tb* strain such as *M. tb* H_37_Ra, induces more apoptosis in macrophage as compared to *M. tb* H_37_Rv virulent strain which evades TNF-dependent apoptosis [62]. Interestingly, our results showed that Rv2231c was a potent suppressor of macrophage apoptosis.

Rv2231c potentiates reactive oxygen species generation in macrophages infected with *Ms*_Rv2231c compared to *Ms*_Vc. However, recombinant Ms_Rv2231c efficiently suppressed RNS production, thereby increasing the survival of macrophages. These results demonstrate that *M. tb*-Rv2231c enables survival in the unfavourable ecosystem within the host macrophages using this intracellular dwelling place to counter the antibody onslaught and maintain timely proliferation. Rv2231c is a remarkable virulence marker of *M. tb*, as evidenced by the higher survival of recombinant *Ms*_Rv2231c within the macrophages.

The structural and functional characterization of Rv2231c as *M. tb* HspAT opens new frontiers for developing *M.tb*-specific drugs. Our study revealed the missing enzyme link in the *M. tb* histidine biosynthetic pathway and extended our current mechanistic understanding of histidine production in *M. tb*. Since Rv2231c is essential for intracellular survival of *M.tb*, biochemical characterization of Rv2231c can aid in developing potent drugs against TB. Our biophysical data provides evidence that Rv2231c is a stable, folded, spherical monomeric protein with a melting temperature of approximately 62 °C.

**Fig. 8:**
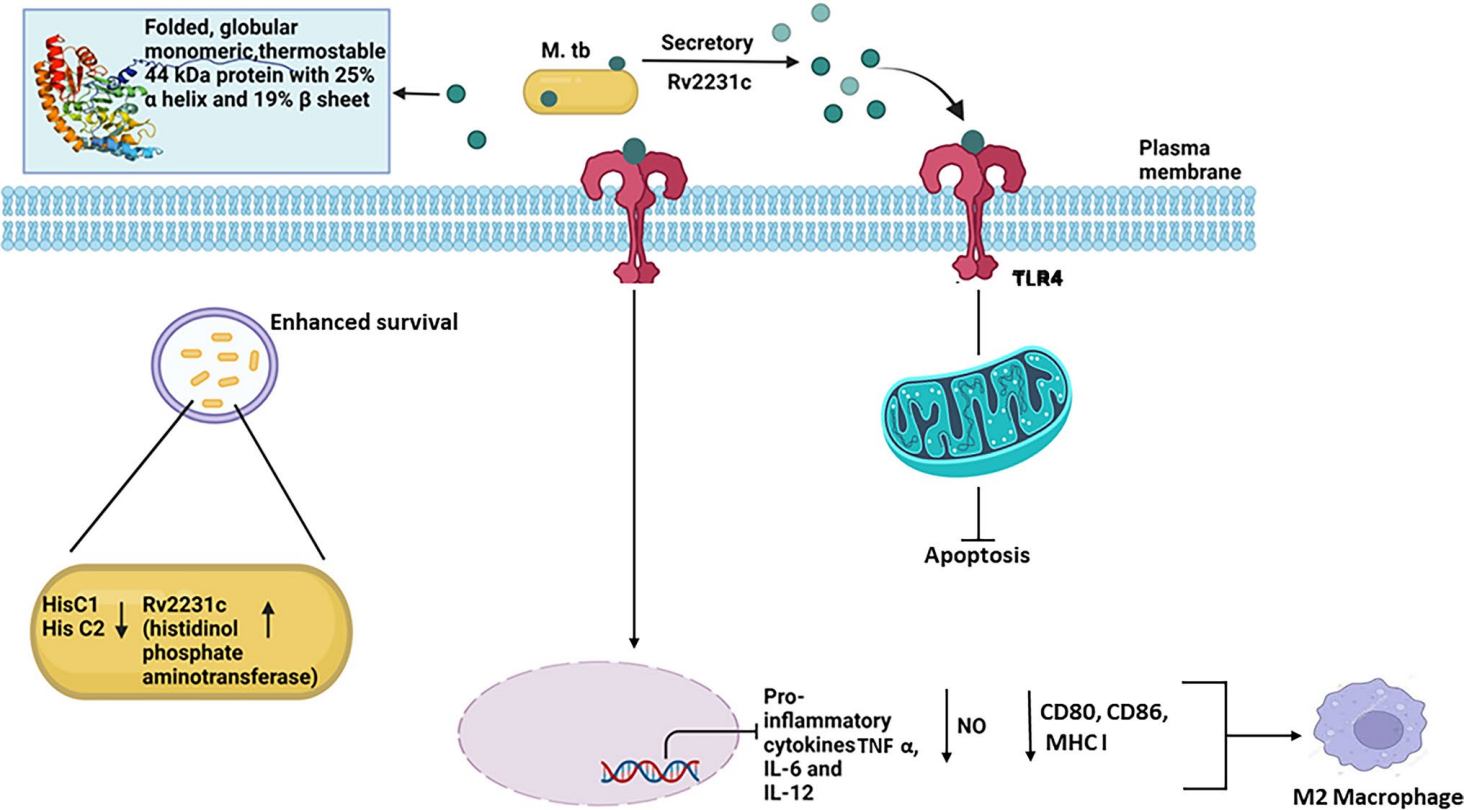
*M. tb* Rv2231c is recognized by TLR4 receptor to regulate downstream signaling cascades. This figure illustrates a summary of the possible mechanism of action *M. tuberculosis* Rv2231c protein

In conclusion, we have provided evidence that *M. tb* Rv2231c acts as HspAT. We hypothesize that HisC1/HisC2 expression decreases and Rv2231c expression increases during macrophage infection, implying that Rv2231c plays a significant role in mediating histidine biosynthesis during *M. tuberculosis* infection in the infected host. A model explaining the action of this protein is depicted in Fig. 8. Rv2231c is a well-folded globular monomer secretory protein that binds with TLR4 present on the surface of macrophage cells, a crucial component of innate immunity. Rv2231c inhibits the secretion of proinflammatory cytokines, which are key antimicrobial molecules. Interestingly, Rv2231c inhibited the expression of macrophage activation markers that induce the propathogen's M2 phenotype. It also dampens the formation of antimicrobial NO, which is critical for immunity against pathogens. Rv2231c enhances virulence by inhibiting host intrinsic apoptosis, thereby promoting the survival of *M. tb*. Our results indicate that Rv2231c is a multifunctional protein from *M. tb* that is exploited to dampen host-directed defence strategies against the pathogen. However, further studies are warranted to extrapolate our results and unravel the switch that regulates the overlapping functions of *M.tb*-Rv2231c.

### Electronic supplementary material

Below is the link to the electronic supplementary material.

Supplementary file1 (TIF 360 KB)

Supplementary file2 (TIF 582 KB)

Supplementary file3 (TIF 424 KB)

Supplementary file4 (TIF 236 KB)

Supplementary file5 (DOCX 28 KB)

Supplementary file6 (PDF 780 KB)

Supplementary file7 (FCS 3520 KB)

Supplementary file8 (FCS 3520 KB)

Supplementary file9 (FCS 3520 KB)

Supplementary file10 (FCS 3520 KB)

Supplementary file11 (FCS 3520 KB)

Supplementary file12 (FCS 3520 KB)

Supplementary file13 (FCS 3520 KB)

Supplementary file14 (FCS 3520 KB)

Supplementary file15 (FCS 3520 KB)

Supplementary file16 (FCS 3520 KB)

Supplementary file17 (FCS 3520 KB)

Supplementary file18 (FCS 3520 KB)

Supplementary file19 (FCS 3520 KB)

Supplementary file20 (FCS 3520 KB)

Supplementary file21 (FCS 3520 KB)

Supplementary file22 (WSP 899 KB)

## Data Availability

All data are provided in the article or in its supplementary materials and are available from the corresponding author on reasonable request.
