# Supplementary figures and images for: Rv2231c, a unique histidinol phosphate aminotransferase from Mycobacterium tuberculosis, supports virulence by inhibiting host-directed defense
Source: Cell Mol Life Sci. 2024 May 2;81(1):203. doi: 10.1007/s00018-024-05200-8 (PMC11065945; doi:10.1007/s00018-024-05200-8)

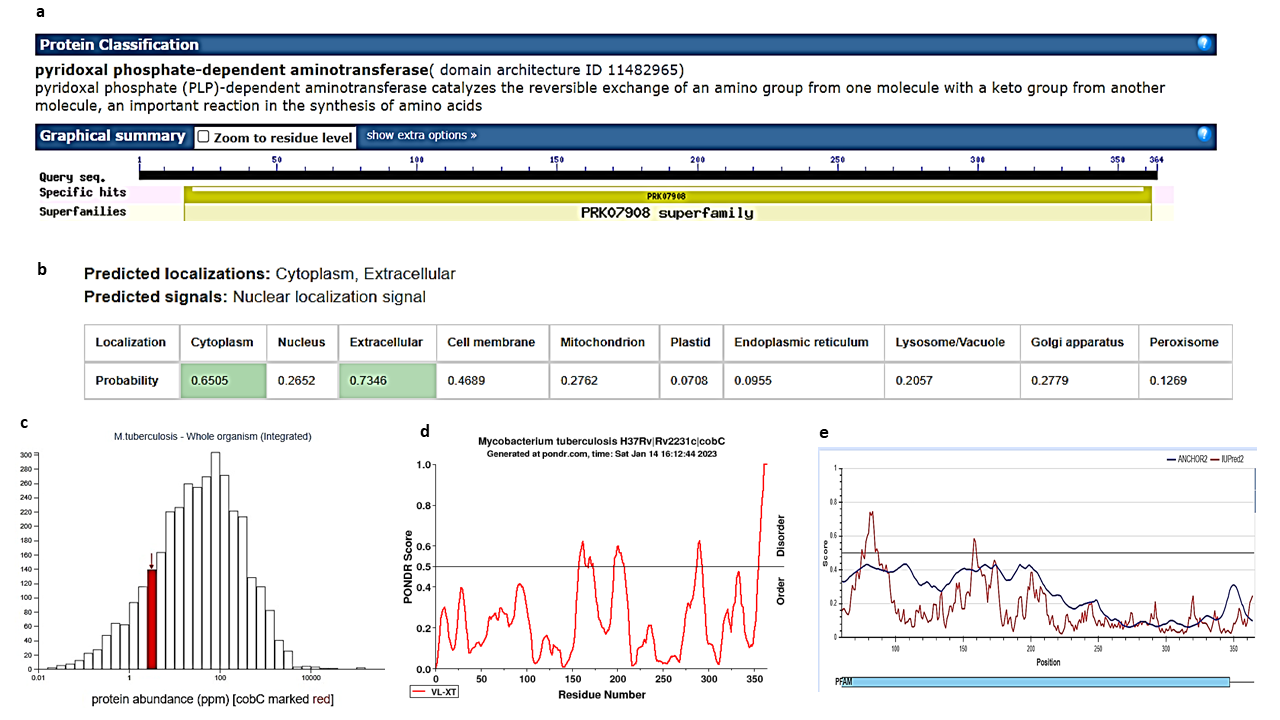

Supplement: Supplementary file 1 — Supplementary file1 (TIF 360 KB) [file 18_2024_5200_MOESM1_ESM.tif]

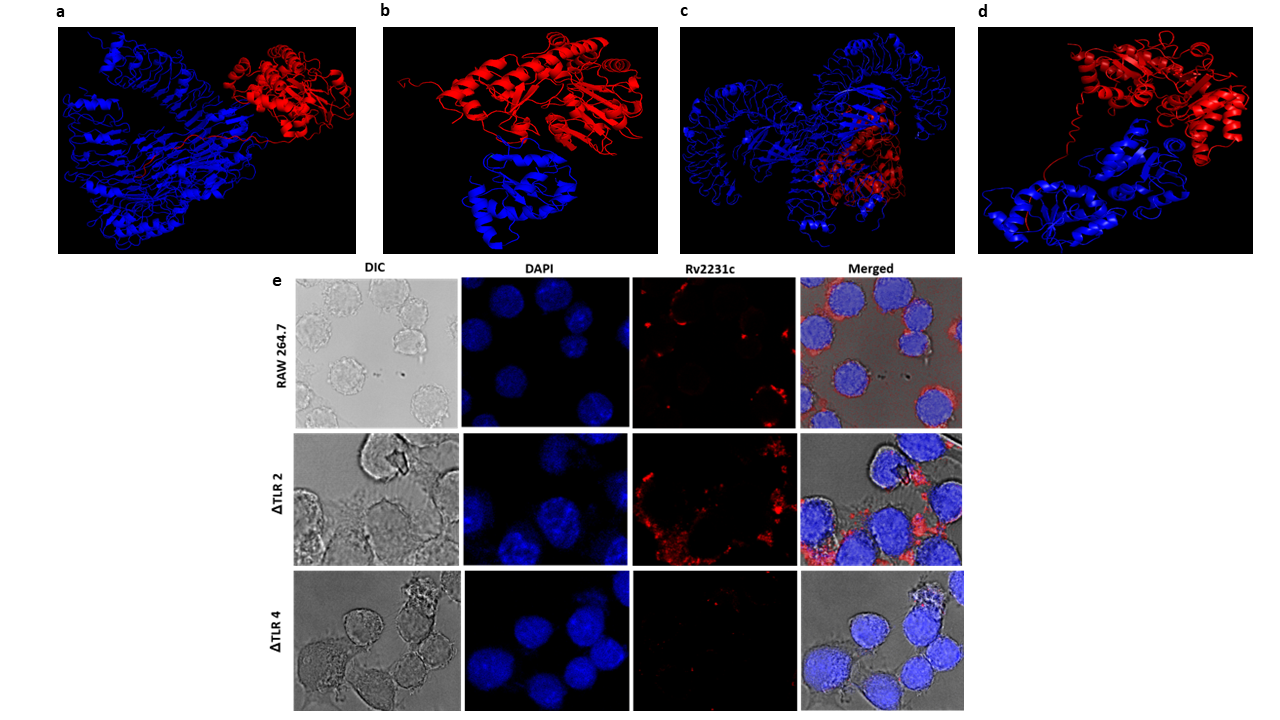

Supplement: Supplementary file 2 — Supplementary file2 (TIF 582 KB) [file 18_2024_5200_MOESM2_ESM.tif]

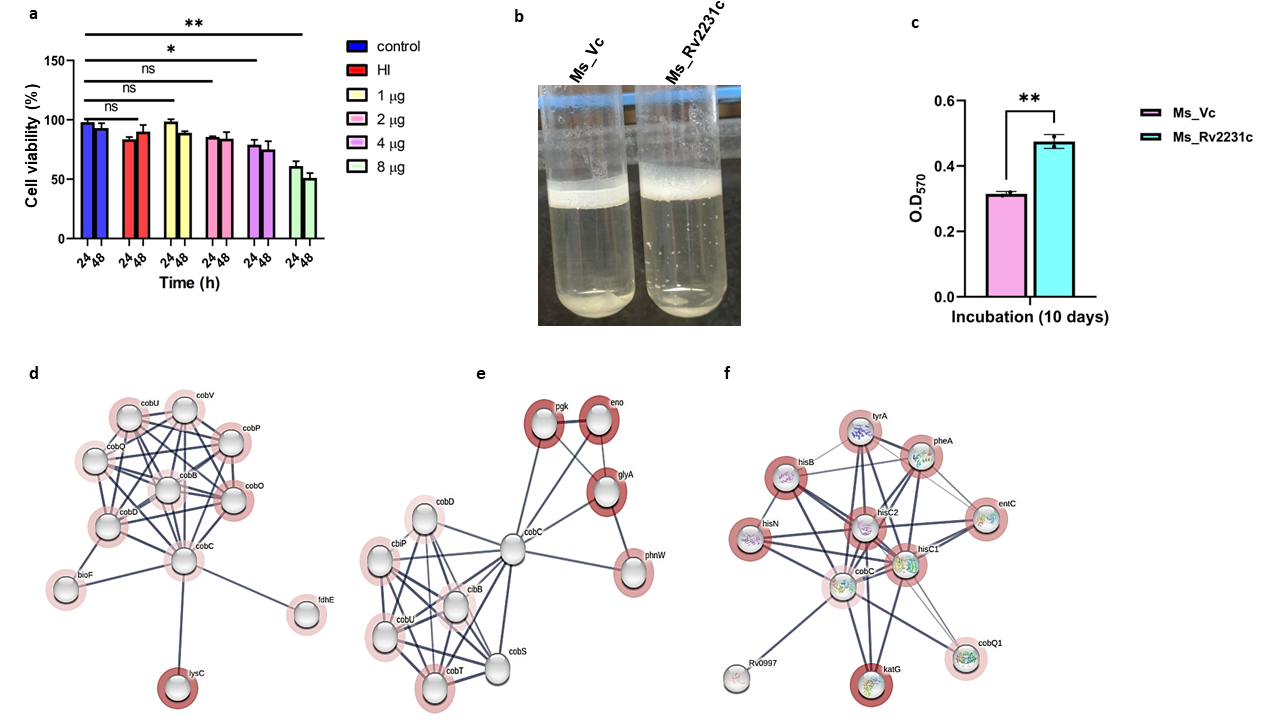

Supplement: Supplementary file 3 — Supplementary file3 (TIF 424 KB) [file 18_2024_5200_MOESM3_ESM.tif]

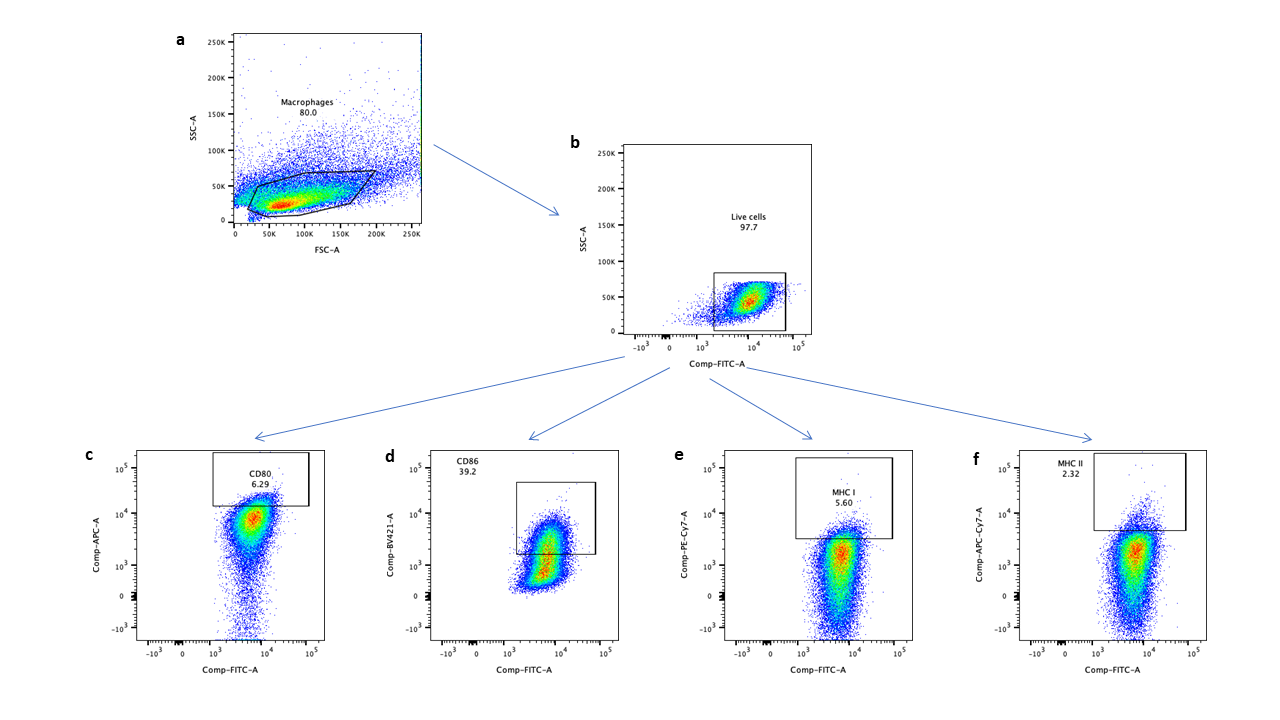

Supplement: Supplementary file 4 — Supplementary file4 (TIF 236 KB) [file 18_2024_5200_MOESM4_ESM.tif]
