# Supplementary material for: Rv2231c, a unique histidinol phosphate aminotransferase from Mycobacterium tuberculosis, supports virulence by inhibiting host-directed defense
Source: Cell Mol Life Sci. 2024 May 2;81(1):203. doi: 10.1007/s00018-024-05200-8 (PMC11065945; doi:10.1007/s00018-024-05200-8)
Supplement: Supplementary file 5 — Supplementary file5 (DOCX 28 KB) [file 18_2024_5200_MOESM5_ESM.docx]

**Supplementary Figures**

**Supplementary Figure 1: Rv2231c contains a (PLP)-dependent aminotransferase domain.** (**a**) Conserved domain prediction using the PROSITE software. (**b**) Prediction of subcellular protein localization using DeepLoc 2.0. (c) Analysis of the relative abundance of *M. tuberculosis* Rv2231c protein (PaxDB 4.0). Rv2231c is among the bottom 25% of the most highly expressed proteins in *M. tuberculosis*. (**d**) Instability index of the Rv2231c protein predicted using PONDR vslr and (**e**) IUPred software.

**Supplementary Figure 2: Rv2231c interacts with the ligand-binding site of TLR4.** (**a–d**) Molecular docking method was used to show the interaction between Rv2231c and TLRs (TLR1, TLR2, TLR4, and TLR6). (TLR4)2-(Rv2231c) top scoring structural model obtained by ClusPro protein-protein docking. (**e**) Recombinant Rv2231c was added to coverslip-grown RAW264.7, TLR2, and TLR4 macrophage cells. Fixed cells were probed with polyclonal antibody against Rv2231c (1:3000) for 2 h. After three washes, the cells were probed for 1 h with anti-rabbit antibody conjugated with Alexa Fluor-594. After three washes, the cells were treated with Prolong Antifade Glass Mount and visualized using confocal microscopy.

**Supplementary Figure 3:** (**a**) RAW264.7 cells were treated with purified *M.tb*-Rv2231c protein (1, 2, 4, and 8 µg/ml) for 48 h, and viability of cell was measured using the MTT assay. Untreated and heat inactivated Rv2231c (HI)-treated cells served as controls. The viability of Rv2231c treated cells was analyzed and compared with controls and presented as percent survival. Rv2231c expression in recombinant *M. smegmatis* induces pellicle formation**.** Sauton’s minimal medium was inoculated with *Ms*_Vc and *Ms*_Rv2231c at 0.1 O.D in glass tubes and incubated under static conditions at 37 °C for 10 days. (**b**) The pellicle produced at the end of incubation. After incubation, the pellicle was estimated using the 0.1% crystal violet method and measured spectrophotometrically at 570 nm (**c**) STRING analysis of Rv2231c proteins from different microorganisms. Interacting partners of Rv2231c from (**d**) [*P. aeruginosa*](https://pax-db.org/species/208964) **, (e)** [*Salmonella enterica subsp. enterica serovar Typhimurium*](https://pax-db.org/species/99287), **(f**) [*M. tuberculosis* H_37_Rv](https://pax-db.org/species/83332).

**Supplementary Figure 4:** Gating strategy for (**c**) CD 80 (**d**) CD 86 (**e**) MHC I and (**f**) MHC II.
