# Supplementary material for: Rv2231c, a unique histidinol phosphate aminotransferase from Mycobacterium tuberculosis, supports virulence by inhibiting host-directed defense
Source: Cell Mol Life Sci. 2024 May 2;81(1):203. doi: 10.1007/s00018-024-05200-8 (PMC11065945; doi:10.1007/s00018-024-05200-8)
Supplement: Supplementary file 6 — Supplementary file6 (PDF 780 KB) [file 18_2024_5200_MOESM6_ESM.pdf]

Table S1: List of primers used in the study.

Primer name	Nucleotide sequence (5' ⟶ 3')	Comment
pET28a-Rv2231c–F	AAGGATCCATGGTGCTCTGGATTCTTG	Cloning of Rv2231c into pET28a and pSTKi vectors
pET28a-Rv2231c–R	AAGAATTCCACACTCATCGGCGTCCTC	

Table S2: The concentration of reactants used for assaying Rv2231c transaminase activity.

Constituents	Concentrations
Tris buffer (pH-8.0)	50 mM Tris-HCL, 150 mM NaCl
NAD+	250 mM
Pyridoxal-5-phosphate	20 µM
Histidinol phosphate	2000 µM
2-Oxoglutarate	1000 µM
Glutamic dehydrogenase	2.37 U/ml
Enzyme (Rv2231c)	1 µM

NAD^+; nicotinamide adenine dinucleotide.

Table S3: Kinetic parameters of *M. tuberculosis* Rv2231c.

Kinetic parameters (amino transferase activity)	Result
K_M [mM]	0.6 ± 0.05
K_{cat} [S^{-1}]	(4.33±0.07) × 10^2
K_{cat} /K_M [$M^{-1}S^{-1}$]	(0.72±0.02) × 10^6

Table S4: Secondary structure of Rv2231c.

S.No	Protein	% α-helix		% β-sheets	
		CD	FTIR	CD	FTIR
1	Rv2231c	28.84	25	13.66	19

CD; circular dichroism, FTIR; fourier transform infrared spectroscopy.

Table S5: Tm of Rv2231c determined using fluorescence, CD, and DSC.

S.No	Protein	CD	DSC
1	Rv3221c	55˚C	67˚C

CD; circular dichroism, DSC; differential scanning calorimetry.
